# Supplementary material for: Third-generation cephalosporin resistant Escherichia coli in dogs and cats in Germany in 2019–2021
Source: PLoS One. 2024 Aug 26;19(8):e0309554. doi: 10.1371/journal.pone.0309554 (PMC11346920; doi:10.1371/journal.pone.0309554)
Supplement: Attachment — Submitted filename: Review no2.docx [file pone.0309554.s002.docx]

Dear Authors,

Thanks for uploading a new manuscript version. The manuscript improved with the modifications.

Nevertheless, there a recommendation on the manuscript:

**Abstract:**

Method-Section:

Line 37-38: you can do AST of bacterial strains/isolates but not from a sample, maybe “AST form isolates obtained from samples collected…”?

Line 40: the number of E.coli isolated is not relevant for the paper; you provide results for 25.404 E. coli with an MIC, so this should be the number in the abstract, as later in the abstract, the rates given were calculated based on this

Result part: question on the rates, please see in the main text review

Conclusion part: Line 52-55 I would suggest to re-write the sentence e.g. …to their owners and transmission of 3GCR between them is likely as well as acquisition from other (environmental) sources. [Comparison to human data here is too separated, as there can’t be an discussion on this fact here and as you do not know about the bias of pre-treatments, duplicates etc…]

**Introduction:**

**Line 68-70:** I would shift this to the end of the first paragraph, it is here off-context and fits better to the transmission mentioned above.

Method:

Line121: “**p**reparation” (small letters)

**Results:**

In- and exclusion criteria for the study should be part of the method section as well as number of isolated E. coli per sample. Further you should exclude all isolates for analysis of all microbials of which data is missing (see figure 3). There are inconsistencies in the number resulting E. coli with accessible MIC; if there are 87 samples with two obviously different E. coli, they count double in the numbers of assessable MIC, so 25.491? The problem here is, that you count samples, but this is the number of E. coli isolates, which makes it difficult to follow also in Table 1. It would be easier to use the number of isolates consequently. Last three lines are confusing, wouldn’t bring them in the table. Just explain in the text, that there are 87 samples with two different E. colis variants isolated and what was done (used both or just one, as your numbers indicate) [all other samples just one E. coli was picked

Figure 2 makes only sense, if the information is connected to something else. Land use, agriculture, population density, WWTP or whatever; as information is not assessable from ARS, you should proof more than one hypothesis.

Line 186-188 (and Fig3): Numbers to compare rates should be only on isolates where you have all MIC information. Different numbers are confusing and are not useful to compare rates. Further you should compare resistance rates of other antimicrobials than cefovecin between 3GCR and on-GCR. It makes no sense to compare rates of 3GRC with a population including the same isolates. (abstract as well)

**Discussion:**

Second paragraph (comparability of FOV to other 3GC) should be moved after the first sentence of the first paragraph. Rest of the first paragraph fits better to the third one.

In both paragraphs (actually 1 and 3) you just mention other publications and numbers but a real discussion is missing. Are the different reports comparable (interpretation breakpoints vs ECOFF)? Are there information on pre-treatments and so on given? etc.

Line 206/207: suggest to re-write the sentence; CTX-M and CMY were detected, doesn’t mean that other resistance mechanism (TEM, SHV) wouldn’t mediated cefovecin resistance (Ref 24 detected them) e.g. “Cefovecin resistance was detected in ESBL as well as AmpC-producing E. coli…”

In general: you mention OneHealth but only focussed on humans (weak discussion on human ESBL rates, by the way); there is no real discussion on environment, livestock and food (raw meat especially for dogs a definite possible source). Tip: by comparing your data (resistance rates) with literature, be sure to use data from the same isolation procedure; do not compare selective isolation (e.g. ESBL/AmpC monitoring), instead have a look on indicator E. coli monitoring / studies. But, as selective isolation result in higher detection rates, don’t forget to discuss this and the meaning for your data.

Line 218: Paragraph on GermVet here and also later in the discussion; suggest to combine both to avoid redundancies

Line240: a) please use the original reference, this is a book chapter, which should have a refence list (same line 258)

b) although there is a transmission to environment including soil, the sentence suggests that there is a higher contamination rate in soil and therefore dominant suggested source. Please provide appropriate original research reference and discuss further points (see above)

Line 244-249: Paragraph out of context, could be integrated to second paragraph for comparability of FOV to other 3GC

Line 250-255: see above; an in depth discussion is missing, otherwise this part and figure can be deleted;

Line 262/263: Last sentence has to be revised

Line 269: re-write e.g. therefore, detailed and current data in AMR rates is needed…

CAZ: in discussion and conclusion: although you introduced CAZ as abbreviation, whole word would be easier to follow for the reader, especially as there are several abbreviations for ceftazidime used between sectors and methods…
